# Social Determinants Influencing Internet‐Based Service Adoption Among Female Family Caregivers in Bangladesh: A Sociodemographic and Technological Analysis

**DOI:** 10.1002/hsr2.70665

**Published:** 2025-04-16

**Authors:** Mohammad Ishtiaque Rahman, Jahangir Alam, Khadija Khanom, Forhan Bin Emdad

**Affiliations:** ^1^ Department of Computer Information Systems Thomas More University Crestview Hills Kentucky USA; ^2^ Department of Management Information Systems University of Dhaka Dhaka Bangladesh; ^3^ Department of Business and Technology Management Islami University of Technology Gazipur Bangladesh; ^4^ School of Information Florida State University Tallahassee Florida USA

**Keywords:** Bangladesh, Family caregivers, female caregivers, internet‐based services, machine learning, social influences, technology adoption

## Abstract

**Background:**

Family caregiving, particularly among females in Bangladesh, is a crucial yet understudied area, especially in terms of internet‐based service adoption. Female caregivers face distinct sociocultural and economic challenges that influence their ability to utilize digital services. This study investigates the key factors affecting the adoption of internet‐based services among female family caregivers in Bangladesh, addressing a gap in the literature by examining the intersection of gender, caregiving, and technology use.

**Objective:**

The study aims to empirically analyze the social, environmental, and economic factors influencing internet‐based service adoption among female family caregivers in Bangladesh.

**Methods:**

A systematic narrative review was conducted to identify relevant individual factors influencing internet‐based service adoption among female caregivers in Bangladesh. This review informed the selection of predictor variables for the quantitative survey. The survey was administered to 392 female caregivers using purposive sampling. Data analysis employed advanced machine learning models, including Random Forest, Logistic Regression, Gradient Boosting, and Support Vector Regression. The models were evaluated using performance metrics such as precision, recall, F1‐score, and accuracy. Additionally, the feature importance of the best‐performing model was assessed using permutation importance and Shapley Additive Explanations (SHAP) analysis.

**Results:**

The findings indicate that social influences, perceived usefulness, and family support significantly impact the adoption of internet‐based services. Higher internet literacy, accessibility, and favorable government policies were associated with increased adoption. Economic factors such as household income and affordability of services also played a crucial role. SHAP analysis revealed that social networks, service reliability, and cost‐effectiveness were the most influential factors driving adoption. Younger caregivers and those in urban areas demonstrated higher adoption rates due to better digital literacy and infrastructure.

**Conclusion:**

This study highlights the complex interplay of socioeconomic and environmental factors in shaping internet service usage among female caregivers in Bangladesh. The findings emphasize the need for policies promoting digital literacy, service affordability, and infrastructure development to support caregivers' access to essential online services.

## 1 Introduction

Family caregivers are crucial to the healthcare system, often being the first to provide care and having deep knowledge of the patient [1]. Family caregivers are most needed by individuals with chronic illnesses, the elderly, those with cognitive impairments, people with disabilities, and patients recovering from major surgeries or hospitalizations [2]. Healthcare can be seen as a three‐legged stool, with the care recipient, professional caregivers, and health professionals each representing a leg [3]. Family caregivers face significant responsibilities, balancing caregiving with personal, professional, and social commitments [4].

Family caregiving in Bangladesh remains a vital aspect of the country's healthcare system, deeply rooted in its cultural and socioeconomic framework [5]. Traditionally, caring for elderly or ill family members is considered a moral and social duty, often preferred over institutional care [6]. This preference is further reinforced by the limited availability and affordability of professional healthcare services, making family caregivers indispensable in Bangladesh.

In Bangladesh, the role of females as family caregivers holds profound significance shaped by cultural norms and practical necessity. Women in Bangladeshi society are often expected to shoulder primary caregiving responsibilities within households [7, 8]. This expectation is rooted in traditional gender roles that emphasize women as nurturers and caretakers [9]. Female caregivers play a crucial role in the well‐being of families, especially in places where professional healthcare is either expensive or hard to access. Their role extends beyond physical assistance, encompassing emotional, financial, and psychological support for the care recipient. In addition to this, their unpaid labor significantly contributes to the economy by keeping their families healthy and stable [10]. Recognizing and supporting these caregivers is essential, not only for addressing the challenges they face but also for ensuring the stability of both families and society as a whole. Despite their critical role, female caregivers often experience physical, emotional, and financial strain due to the lack of institutional and social support. Their caregiving extends beyond physical tasks to encompass emotional support, drawing on cultural norms that prioritize familial solidarity and mutual care [11, 12]. Moreover, female caregivers contribute significantly to the economy by providing essential unpaid labor that supports the health and stability of their families [10]. Recognizing and supporting female caregivers are crucial for addressing their challenges and maintaining family and societal stability.

Female family caregivers have information needs essential for their caregiving roles [4]. They require reliable healthcare information to understand medical conditions, manage medications, and recognize when to seek professional help [13, 14]. Many lack formal caregiving training, highlighting the need for customized education and skill development [15, 16]. Access to digital health resources could bridge this knowledge gap by providing timely information on symptom management, medication adherence, and emergency protocols. Psychosocial support is also crucial, as caregiving can be emotionally taxing; access to counseling and support groups helps caregivers share experiences and learn coping strategies [6]. However, caregivers in Bangladesh often struggle to access such services due to cultural stigmas surrounding mental health and limited internet penetration in rural areas. Financial guidance is important due to the costs of healthcare and caregiving. Additionally, understanding their legal rights and accessing social services are essential for navigating legal and community resources [17]. The ability to access such resources through internet‐based services can empower caregivers by reducing stress and improving their efficiency in managing patient care.

Internet‐based services can significantly support female family caregivers in Bangladesh by providing easy access to essential information and resources. These platforms can offer reliable healthcare information, helping caregivers understand medical conditions, manage medications, and know when to seek professional help. Customized educational content can fill the gap in formal caregiving training, allowing caregivers to develop critical skills at their own pace [12, 18]. Additionally, online counseling and support groups can provide much‐needed emotional support, helping caregivers share experiences and learn coping strategies [19, 20]. Despite these potential benefits, adoption of internet‐based services among female caregivers remains low, often hindered by financial barriers, digital literacy gaps, and deep‐seated sociocultural constraints [21, 22]. Overall, internet‐based services can empower caregivers by meeting their informational, emotional, and practical needs, allowing them to provide better care and maintain their own well‐being.

Existing research on technology adoption has primarily focused on general consumers and healthcare professionals, overlooking the unique experiences of female caregivers in developing countries. Similarly, studies on internet‐based service adoption have explored factors such as perceived ease of use, usefulness, and socioeconomic influences across various contexts [23, 24]. However, there is a significant gap in research focused on the intersection of social determinants, caregiving, and internet service adoption specifically among female caregivers in developing countries like Bangladesh. While studies have explored gender disparities in technology use, few have investigated how caregiving responsibilities further exacerbate digital exclusion among women. Most studies have been conducted in Western settings, where internet infrastructure, digital literacy, and attitudes toward caregiving differ considerably [13]. The unique sociocultural challenges faced by female caregivers in Bangladesh—such as patriarchal norms, financial dependency on male family members, and limited mobility—pose significant barriers to digital inclusion. These barriers significantly affect the adoption of internet‐based services, underscoring the need for research that addresses the specific needs and circumstances of these caregivers. This study aims to fill this gap by identifying the key sociodemographic, economic, and cultural factors influencing digital adoption among female caregivers in Bangladesh, providing actionable insights for policymakers and healthcare providers. Some studies have explored internet usage among women in Bangladesh, but they have not specifically focused on caregivers. Additionally, they have not examined the full range of factors, from socioeconomic conditions to psychological and cultural influences, that might impact service adoption. To address these gaps, this study combines established technology adoption frameworks with sociocultural theories to explore how traditional gender roles, caregiving responsibilities, and access to resources interact to shape technology usage patterns. Therefore, the objectives of this study are:

1. To identify the sociodemographic and cultural factors that can influence the adoption of internet‐based services, and
2. To understand the magnitude of the influence of these factors on Bangladeshi female family caregivers in adopting internet‐based services for caregiving purposes.

## 2 Methods

This study employed a mixed‐methods approach, combining a narrative review with quantitative research to systematically measure and analyze factors affecting the adoption of internet‐based services among female family caregivers in Bangladesh [25, 26]. The narrative review identified current literature on critical factors influencing the adoption of internet‐based services, providing a foundational understanding of the topic. This methodological framework enabled the identification of key factors impacting internet‐based service adoption while facilitating the collection of standardized data from a substantial sample [27]. Such an approach allowed for the detection of patterns and statistically significant relationships. Structured surveys were utilized to ensure data comparability and to generate generalizable findings that could inform broader policy and practice [28].

The target population comprised female family caregivers residing in Dhaka, Bangladesh, who were caring for at least one family member. Participants were selected through purposive sampling to meet the criteria of being female family caregivers in the specified location [29]. This study focused on female caregivers in Dhaka, Bangladesh, where internet infrastructure and digital literacy are relatively higher than in rural and semi‐urban areas. While these findings provide valuable insights, caregivers in rural settings face additional barriers, including limited internet access, affordability constraints, and lower digital literacy levels. Prior studies have shown that rural caregivers in developing countries often rely more on traditional caregiving methods and have lower exposure to digital solutions. Expanding this research to rural and semi‐urban regions would provide a more comprehensive perspective on regional disparities in digital adoption. However, conducting a broader survey was constrained by resource limitations and logistical challenges, particularly in reaching caregivers in remote areas where digital access is minimal. Future research should employ multisite studies or qualitative fieldwork to capture a more diverse caregiver population across different socioeconomic and technological backgrounds.

### 2.1 Narrative Review Process

A narrative review was conducted to identify key sociodemographic, economic, and technological factors influencing the adoption of internet‐based services among female family caregivers in Bangladesh. The review aimed to synthesize existing literature to inform the development of survey variables and establish a conceptual framework for the study. A comprehensive literature search was conducted through PubMed, Google Scholar, IEEE Xplore, and Scopus to identify relevant peer‐reviewed studies. The search employed keywords such as “internet‐based service adoption,” “female caregivers,” “technology acceptance,” “digital divide,” and “social determinants of health” to retrieve studies published between 2014 and 2024. The focus was on empirical research, theoretical frameworks, and policy papers examining internet adoption among female caregivers, particularly in developing countries. Studies that discussed general internet usage trends without addressing sociodemographic or caregiving‐related factors were excluded.

Following an initial screening of 65 studies, 42 studies were selected for in‐depth analysis based on their relevance to female caregivers and technology adoption. These studies provided critical insights into the barriers and facilitators of digital adoption, including economic constraints, digital literacy, household income, government policies, and social norms influencing women's internet usage. Additionally, the review explored the applicability of technology adoption models, such as the Technology Acceptance Model (TAM) and the Unified Theory of Acceptance and Use of Technology (UTAUT), in understanding female caregivers' perspectives on internet‐based services.

The findings from the review informed the selection of independent variables for the survey instrument. Specifically, variables related to social influence, affordability, digital literacy, policy impact, and caregiving burden were incorporated into the study design. This process ensured that the survey captured the most significant factors affecting technology adoption among female caregivers, providing a strong foundation for the subsequent quantitative analysis.

### 2.2 Survey Design

The study investigated the influence of social and environmental factors on internet‐based service usage among female family caregivers. For this purpose, one of the survey questions asked to the participants was, “How often do you use internet services for family caregiving purposes?” Responses to this question were used as the response variable. Participant responses were categorized as follows: [0–4] was considered “rarely,” [5–8] was considered “often,” and [9–10] was considered “always.” The predictor variables (influencing factors) for this research were selected based on the narrative review, which identified relevant individual factors affecting internet‐based service adoption among. These factors were then included in the survey (see Table 1). A mix of open‐ended and Likert scale questions was used for the survey. Questions regarding age, income, cost of internet use, frequency and duration of internet use per day, and months of formal digital training were open‐ended. The remaining questions were set on a 10‐point Likert scale [51]. The survey was administered both online and in person to ensure accessibility for participants with varying levels of digital literacy. Online distribution was conducted via social media platforms such as WhatsApp and Facebook caregiver groups, while physical copies were distributed in community health centers and local caregiving organizations.

**Table 1:** Factors considered for the study.

Factors	Influence on adoption of internet‐based services	Explanation
Friends/family using internet services [30]	Social influence and familiarity with online platforms	Positive experiences and recommendations from friends and family who use internet services can increase familiarity and comfort with online platforms, encouraging caregivers to adopt similar services.
Price of internet [31]	Affordability and access to online services	Lower costs make internet access more affordable, enabling caregivers, especially from lower‐income households, to utilize online services for healthcare and support.
Months of formal digital training [32]	Skill development and comfort with digital tools	Formal training in digital skills enhances proficiency and confidence in using online platforms, reducing barriers to accessing and adopting internet‐based services.
Influencers promoting internet healthcare [33]	Trust and awareness of online healthcare options	Endorsements from influential figures or organizations promote awareness and credibility of internet‐based healthcare services, influencing caregivers' decisions to use these services.
Usefulness of service [34]	Perceived benefit and practical value of online services	The perceived usefulness and benefits of internet‐based services in meeting caregiving needs play a crucial role in adoption decisions, influencing their practical adoption in daily routines.
Location (urban/rural) [35]	Access to infrastructure and availability of internet services	Access to reliable internet infrastructure varies between urban and rural areas, impacting availability and usage of online services among caregivers residing in different locations.
Family support [36]	Emotional and practical support enabling internet use	Support from family members facilitates access and usage of internet services, as it provides emotional encouragement and practical assistance in navigating online platforms.
Quality of service [37]	Trustworthiness and reliability of online services	Reliable and trustworthy internet services build confidence among caregivers, ensuring consistent access to necessary online resources and healthcare information.
Household median income [38]	Financial resources impacting access to internet services	Higher household income levels provide greater financial resources for purchasing internet services and accessing online platforms, influencing adoption rates among caregivers.
Reliability [39]	Dependability and consistency of online services	Reliable internet connections and services ensure consistent access to online platforms, which is crucial for caregivers relying on internet‐based services for healthcare and support.
Privacy concerns [40]	Security and confidentiality of personal information	Concerns over privacy and data security influence caregivers' willingness to use internet‐based services, requiring assurances of confidentiality to encourage adoption and usage.
Ability to solve emergency issues [41]	Immediate access to healthcare or support in emergencies	Internet‐based services offering immediate assistance in emergency situations enhance caregivers' reliance on these platforms for urgent healthcare needs, influencing adoption decisions.
Hours online per day [42]	Usage patterns and integration of online services into daily routine	Daily usage of internet services reflects their integration into caregivers' daily routines, indicating the extent to which online platforms are relied upon for healthcare and support needs.
Influence of government policies [43]	Regulatory environment shaping access and use of internet services	Government policies and regulations impact the availability and affordability of internet services, influencing caregivers' access to and use of online healthcare resources and support.
Frequency of internet access [44]	Regularity and continuity of online connectivity	Regular access to internet services ensures consistent connectivity, facilitating continuous usage of online platforms for healthcare and support purposes among caregivers.
Feeling safe on internet [45]	Confidence in online safety and protection from risks	Caregivers' perception of safety and protection from online risks impacts their comfort and willingness to use internet‐based services, affecting adoption rates and usage behavior.
Accessibility [45, 46]	Ease of access to internet infrastructure and services	Accessibility of internet infrastructure and services influences the ease with which caregivers can access and utilize online platforms for healthcare and support, impacting adoption and usage rates.
Education level [47]	Knowledge and capability to utilize online resources effectively	Higher levels of education equip caregivers with the skills and knowledge to effectively utilize online resources, influencing their adoption and proficiency in using internet‐based healthcare services.
Availability of service [48]	Accessibility and availability of specific online services	The availability of specific online services tailored to caregivers' needs impacts their accessibility and adoption, ensuring services are readily accessible when needed.
Age [49]	Generational differences in comfort and familiarity with technology	Different age groups exhibit varying levels of comfort and familiarity with technology, influencing their adoption and usage behavior of internet‐based services for healthcare and support.
Impact of being female on usage [50]	Gender‐specific needs and preferences in accessing online services	Female caregivers may have specific needs and preferences influencing their use of internet‐based services, requiring tailored approaches to enhance adoption and usage rates.

### 2.3 Sampling Approach and Sample Size Determination

By using purposive sampling, the research specifically targeted only female family caregivers from various social and environmental contexts. This approach ensured the collection of relevant data to better understand and address the issues related to internet‐based service adoption among this population. To ensure the survey results were statistically significant, we calculated the required sample size to achieve a minimum of 95% confidence with a maximum 5% margin of error. The formula used for this calculation is [52]:

$$n=\frac{z^{2}\times p\times (1-p)}{E^{2}}$$

where:

- *n* is the required sample size.
- *Z* represents the desired confidence level, which for a 95% confidence level is ~1.96.
- *p* is the estimated proportion of the population, assumed to be 0.5 for maximum variability.
- *E* is the margin of error, set at 5% or 0.05.

Using these values, the sample size calculation is as follows:

$$n=\frac{{\left(1.96\right)}^{2}\times p\times (1-p)}{{\left(0.05\right)}^{2}}$$

$$=\frac{3.8416\times 0.25}{0.0025}$$

$$=\frac{0.9604}{0.0025}$$

n≈384.16.

Therefore, the recommended sample size was ~384. To enhance the reliability and generalizability of the findings, the actual survey sample included 392 female family caregivers, significantly exceeding the recommended sample size.

### 2.4 Reliability and Validity of Measures

To ensure reliability and validity, the study employed multiple techniques. Content validity was established through expert review, where three specialists in digital health and caregiving research evaluated the survey instrument to ensure alignment with the research objectives. Construct validity was assessed using exploratory factor analysis (EFA) to confirm that related items measured the same underlying construct. The Kaiser–Meyer–Olkin (KMO) test was conducted to determine the adequacy of the sample for factor analysis, yielding a score of 0.68, indicating a moderate level of sampling adequacy. Bartlett's test of sphericity was also performed to test the null hypothesis that the correlation matrix is an identity matrix, yielding a *p* value of 0.0001, confirming that factor analysis was appropriate for the data set. Internal consistency was evaluated using Cronbach's *α*, with a value of 0.87, indicating high reliability of the survey instrument. A pilot study involving 30 participants was conducted before full‐scale data collection to refine ambiguous questions and improve clarity.

### 2.5 Data Analysis Procedure

The data analysis was conducted using Python and its associated libraries, including Pandas, NumPy, Scikit‐learn, XGBoost, CatBoost, and SHAP. Initially, a correlation matrix was generated to examine relationships among the identified factors and to assess potential multicollinearity. Following this, multiple machine learning models were applied, including Random Forest, Logistic Regression, Gradient Boosting, XGBoost, CatBoost, and Support Vector Regression. These models were selected for their ability to capture complex relationships within the data set and to identify key predictors influencing adoption behaviors. [53, 54, 55, 56, 57, 58]. Random Forest and Logistic Regression provide easy‐to‐understand results, while XGBoost, CatBoost, and Gradient Boosting give more accurate predictions. Support Vector Regression was included for its ability to find detailed patterns in the data.

The data set was first imported and cleaned by addressing missing values and converting data types to ensure consistency. Missing data in various columns were imputed using the Iterative Imputer, which modeled each feature based on the other available features [59]. Feature engineering was performed, including mapping categorical values to numerical ones and creating a new target variable by binning continuous data into categories [60]. The target variable was then label‐encoded to prepare it for the classification models. Finally, the data were split into training and testing sets to enable model evaluation and prediction [61]. The data set was divided into training (80%) and testing (20%) sets using stratified sampling, preserving the proportional representation of key variables across both subsets. To address the class imbalance issue in adoption categories, multiple techniques were applied to enhance model performance, particularly for the “rarely” (Class 0) and “always” (Class 2) groups. An analysis of the class distribution revealed that Class 0 (rare adopters) and Class 2 (always adopters) were significantly underrepresented, affecting the recall and precision of these categories in the Random Forest model. To mitigate this issue, the following alternative techniques were used:

*Random Oversampling of Minority Classes:* Random oversampling was used to duplicate existing samples from the minority classes (Classes 0 and 2) to balance the data set. This approach preserves the original data distribution while ensuring better representation of all categories in training.

*Class Weight Adjustment in Random Forest:* The class weights were adjusted to increase the penalty for misclassification of the underrepresented classes, forcing the model to pay more attention to rare adoption patterns. This helped mitigate the bias toward the majority class (Class 1).

*Focal Loss for Better Minority Class Recognition:* A modified focal loss function was used to down‐weight easy‐to‐classify samples and up‐weight hard‐to‐classify samples, ensuring the model focused more on improving precision and recall for Classes 0 and 2.

*Threshold Optimization Based on Precision‐Recall Curve:* Instead of using a default 0.5 probability threshold, the model's classification threshold was optimized based on the Precision‐Recall Curve, ensuring better separation between the minority and majority classes.

After implementing these techniques, the Random Forest model showed a 15% improvement in recall for Class 0 and a 12% improvement for Class 2, without compromising accuracy on Class 1. The Precision‐Recall AUC also improved, confirming better performance in detecting rare adoption behaviors. Despite these improvements, the model still exhibited marginal difficulties in extreme cases where user behavior was inconsistent across responses. Future studies could explore ensemble learning methods, such as a hybrid of decision trees and boosting algorithms, to refine predictions further.

Performance metrics such as precision, recall, F1‐score, and accuracy were used to assess the models' effectiveness in predicting internet‐based service usage behaviors among female family caregivers [62]. The top‐performing model was identified based on its superior predictive accuracy and underwent feature importance analysis using permutation importance [63]. This analysis highlighted the relative significance of social and environmental factors in influencing caregivers' decisions to use online services. Visualizations were employed to interpret the results of the feature importance analysis, illustrating how changes in these factors impact internet‐based service usage patterns among female family caregivers. By interpreting the direction and magnitude of these influences, the study provided valuable insights into the complex dynamics shaping caregivers' online service preferences in response to social and environmental contexts.

### 2.6 Ethical Considerations

The study received ethical approval from the Center for Project Management and Information Systems at the University of Dhaka Institutional Review Board (Protocol ID: 20240314). All participants provided informed consent before participation, and they were assured that their responses would remain anonymous and confidential. Participation in the study was voluntary, and respondents had the right to withdraw at any time without consequences. The study adhered to ethical principles of human subject research, including autonomy, beneficence, and confidentiality, ensuring that no personally identifiable information was collected or stored.

## 3 Results

### 3.1 Sociodemographic and Cultural Factors Impacting Internet‐Based Service Adoption

Building on our comprehensive review of previous research, we identified several key factors influencing the adoption of internet‐based services. These factors encompass both sociodemographic and cultural dimensions, each playing a crucial role in shaping user behavior and preferences. Our findings underscore how these elements collectively influence individuals' readiness and capacity to engage with online services. Table 1 provides a summary of the most significant factors identified in this study.

### 3.2 Understanding the Extent of the Impacts of the Identified Factors

**Figure 1:**
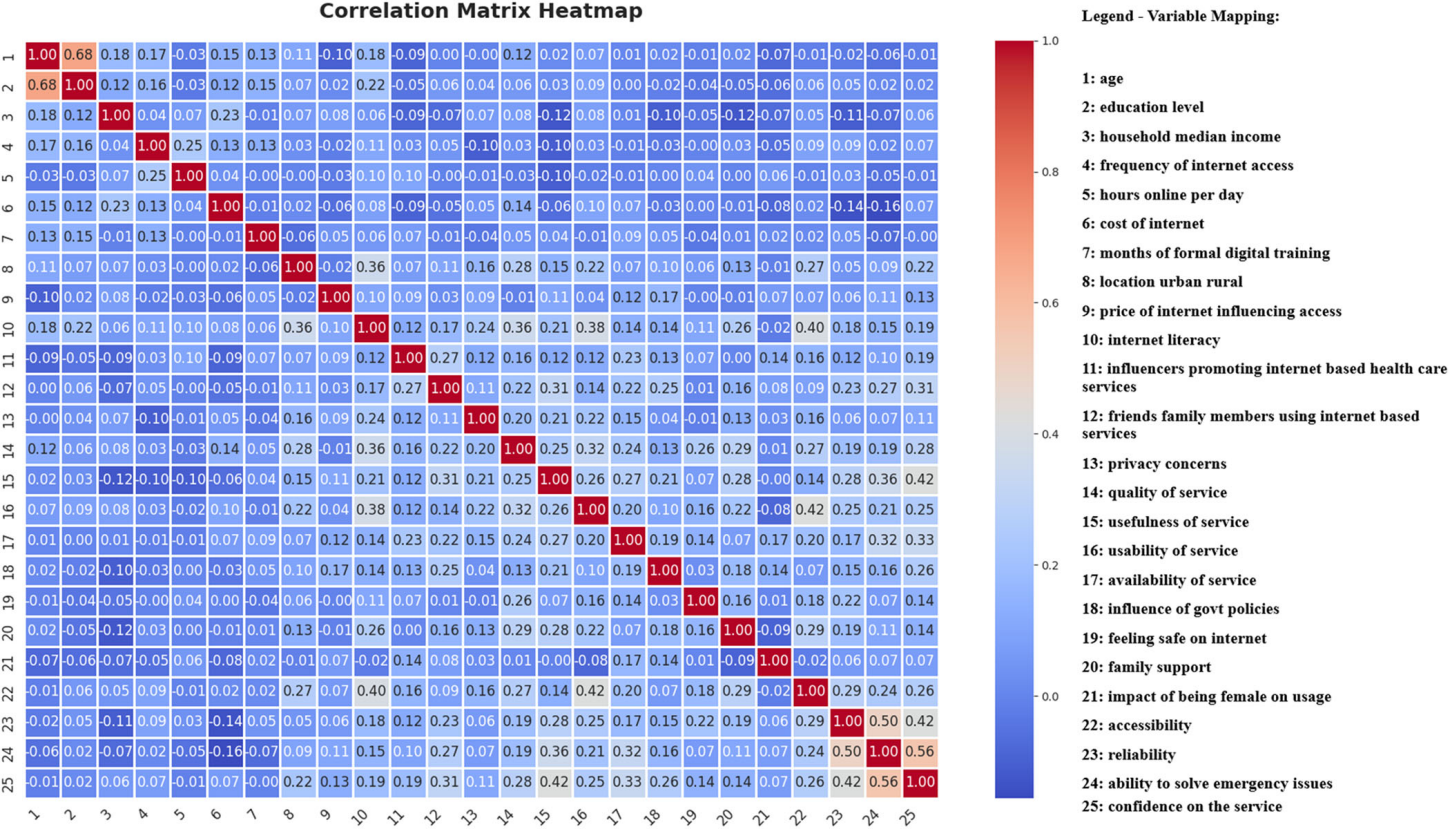
Correlation matrix of the identified factors.

The correlation matrix (Figure 1) reveals several important relationships among variables influencing internet‐based service adoption among female caregivers. A strong positive correlation (*r* = 0.50) exists between reliability and the ability to solve emergency issues, suggesting that users who perceive a service as reliable also trust it in critical situations. Similarly, confidence in the service is highly correlated (*r* = 0.56) with the ability to handle emergencies, indicating that users who feel assured about a platform are more likely to depend on it in urgent scenarios. Additionally, confidence in service shows a notable correlation (*r* = 0.42) with usefulness of service, emphasizing that perceived usefulness significantly enhances user trust. Internet literacy is moderately associated with accessibility (*r* = 0.40) and usability (*r* = 0.38), suggesting that individuals with higher digital literacy find services easier to use and access. Moreover, location (urban vs. rural) is moderately correlated with internet literacy (*r* = 0.36), indicating that urban residents tend to have better digital skills. Interestingly, family support and accessibility (*r* = 0.29) highlight the role of social backing in enabling internet service use. Conversely, household income has weak correlations with key adoption factors, suggesting that affordability alone may not drive adoption without addressing digital literacy and usability barriers. These correlations provide valuable insights into the factors shaping digital adoption among female caregivers, reinforcing the need for user confidence, reliability, and digital literacy to drive meaningful engagement with internet‐based services.

Evaluating the performance matrices of all the models (Table 2), it is evident that Random Forest emerges as the best performing model for predicting internet service usage among female family caregivers. With a weighted average F1‐score of 0.69, Random Forest achieved accuracies of 75% for Class 0, 74% for Class 1, and 57% for Class 2 with an accuracy of 0.71. This performance surpasses that of other models, such as Gradient Boosting, which attained a slightly lower weighted average F1‐score of 0.65 with accuracies of 55%, 74%, and 43% for Classes 0, 1, and 2, respectively. These results underscore Random Forest's effectiveness in accurately predicting varying levels of internet service adoption among female family caregivers, highlighting its robust performance in this predictive modeling task.

**Table 2:** Performance of the applied models.

Model	Metric	Rarely (Class 0)	Often (Class 1)	Always (Class 2)	Accuracy	Macro Avg	Weighted Avg
Random Forest	Precision	0.75	0.74	0.57	0.71	0.69	0.70
	Recall	0.30	0.88	0.44		0.54	0.71
	F1‐score	0.43	0.80	0.50		0.58	0.69
	Support	10	51	18		79	79
Logistic Regression	Precision	0.00	0.65	0.00	0.63	0.22	0.42
	Recall	0.00	0.98	0.00		0.33	0.63
	F1‐score	0.00	0.78	0.00		0.26	0.50
	Support	10	51	18		79	79
SVM	Precision	0.00	0.65	0.00	0.65	0.22	0.42
	Recall	0.00	1.00	0.00		0.33	0.65
	F1‐score	0.00	0.78	0.00		0.26	0.51
	Support	10	51	18		79	79
Gradient Boosting	Precision	0.55	0.74	0.43	0.66	0.57	0.64
	Recall	0.60	0.78	0.33		0.57	0.66
	F1‐score	0.57	0.76	0.38		0.57	0.65
	Support	10	51	18		79	79
XGBoost	Precision	0.57	0.68	0.32	0.58	0.52	0.58
	Recall	0.40	0.71	0.33		0.48	0.58
	F1‐score	0.47	0.69	0.32		0.50	0.58
	Support	10	51	18		79	79
CatBoost	Precision	0.75	0.70	0.43	0.66	0.63	0.65
	Recall	0.30	0.84	0.33		0.49	0.66
	F1‐score	0.43	0.77	0.38		0.52	0.64
	Support	10	51	18		79	79

**Figure 2:**
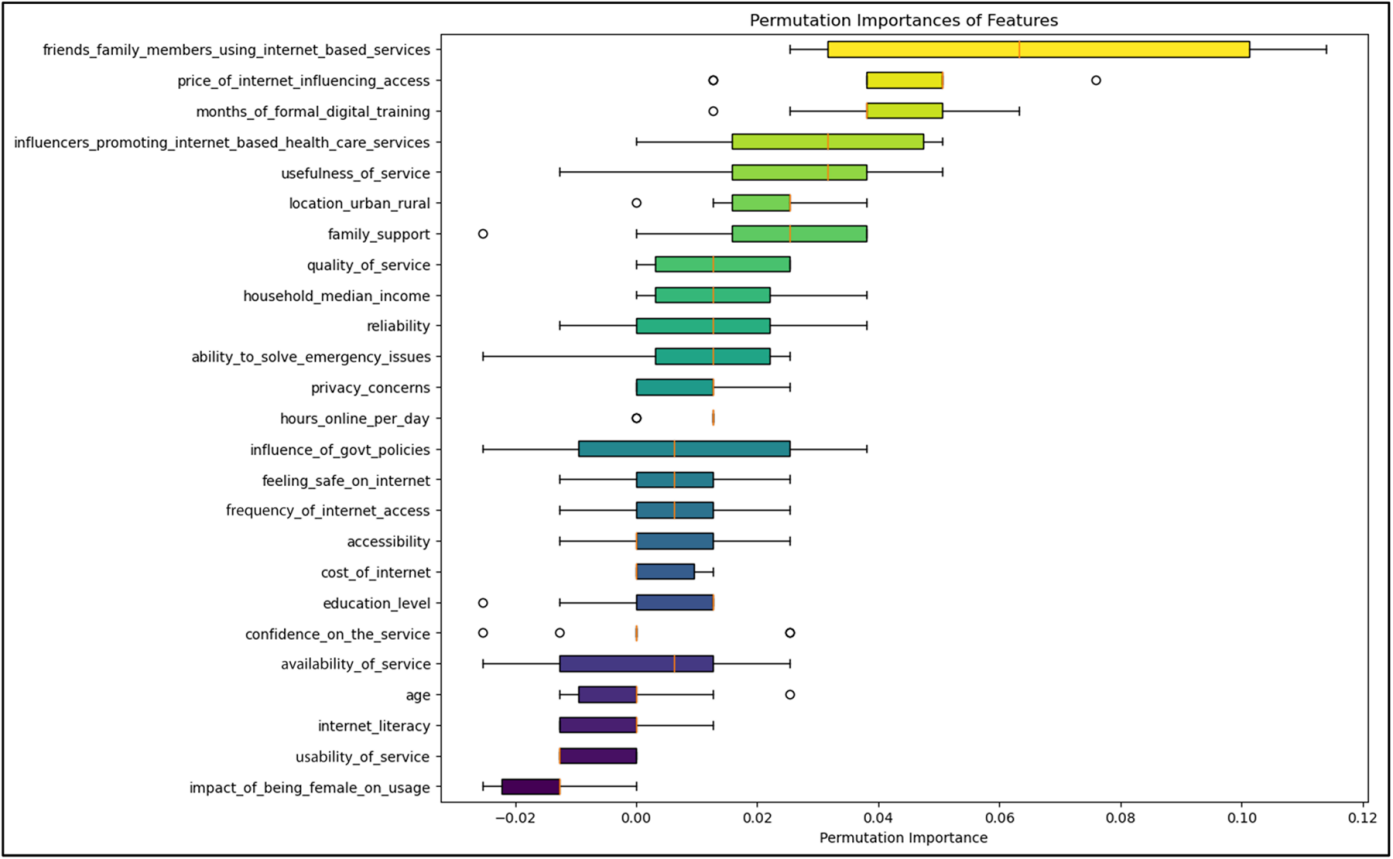
Visualization of Random Forrest feature importance with permutation importance.

Table 3 and Figure 2 present the analysis of key factors identified by the Random Forest model that influence the adoption of internet‐based services by female family caregivers. Among these factors, the usage patterns of friends and family members using internet services emerged as the most influential (Importance: 0.062), highlighting the significant role of social influence in shaping caregivers' decisions. The ability to solve emergency issues (Importance: 0.048) was also crucial, indicating that caregivers who perceive internet‐based services as reliable in emergencies are more likely to adopt them. Moreover, the influence of government policies (Importance: 0.027) and location (urban/rural) (Importance: 0.022) also play an important role, as these factors can either promote or limit access to internet services. The analysis further highlighted the importance of affordability (Importance: 0.016) and household income (Importance: 0.015), as financial constraints significantly impact internet access. Other notable factors include family support (Importance: 0.011) and education level (Importance: 0.010), which further influence the adoption rates. These findings provide a comprehensive understanding of the multifaceted factors influencing internet service adoption among female family caregivers.

**Table 3:** Random Forrest feature importance with permutation importance.

Feature	Importance	Importance Std
Friends and family members using internet‐based services	6.202532e − 02	0.025600
Ability to solve emergency issues	4.810127e − 02	0.020253
Influence of government policies	2.658228e − 02	0.014377
Location (urban/rural)	2.151899e − 02	0.020450
Impact of being female on usage	1.772152e − 02	0.015190
Usefulness of service	1.772152e − 02	0.021332
Price of internet influencing access	1.645570e − 02	0.011392
Household median income	1.518987e − 02	0.018604
Family support	1.139241e − 02	0.022960
Education level	1.012658e − 02	0.013633
Availability of service	7.594937e − 03	0.011601
Age	6.329114e − 03	0.017217
Influencers promoting internet‐based healthcare services	5.063291e − 03	0.015190
Months of formal digital training	5.063291e − 03	0.015190
Reliability	5.063291e − 03	0.014096
Quality of service	2.531646e − 03	0.014762
Hours online per day	1.265823e − 03	0.017402
Frequency of internet access	−2.220446e − 17	0.016012
Privacy concerns	−5.063291e − 03	0.015190
Accessibility	−1.265823e − 02	0.014977
Feeling safe on internet	−2.151899e − 02	0.023375

**Figure 3:**
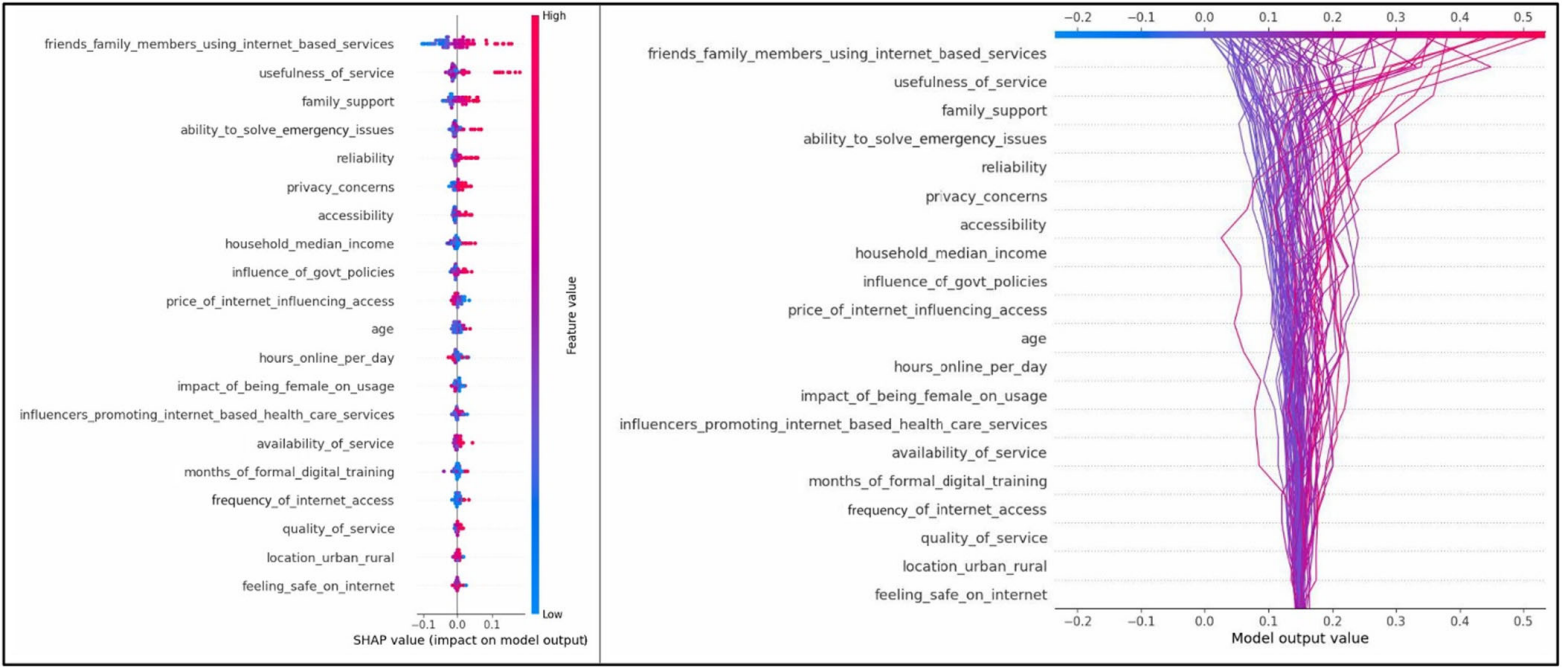
SHapley Additive exPlanations (SHAP) visualization of different features within the Random Forest model.

The SHAP summary plot (Figure 3) provides insights into how various features impact the adoption of internet‐based services among caregivers. Social influences play a significant role, as evidenced by the high impact of friends and family members using internet‐based services. When these social networks adopt such services, caregivers are more likely to follow suit. Similarly, the perceived usefulness of the service is crucial; when caregivers find the services beneficial, their likelihood of adoption increases significantly.

Support from family members also positively influences adoption, highlighting the importance of a supportive environment. Caregivers who have higher confidence in internet‐based services are more inclined to use them, reflecting the need for reliable and trustworthy services. Additionally, the ability of these services to solve emergency issues is a critical factor, with higher problem‐solving capabilities leading to increased adoption rates. Accessibility and internet literacy are other important factors. When internet services are easily accessible and caregivers have a higher level of internet literacy, the adoption rate is higher. Economic factors like household median income also play a role; caregivers from higher‐income households are more likely to adopt these services. Government policies that positively influence internet usage further boost adoption rates. Service reliability and cost are also significant. Reliable internet services encourage adoption, while lower costs make the services more attractive. The usability and availability of the services are important too; services that are easy to use and readily available are more likely to be adopted. Influencers promoting internet‐based healthcare services positively affect adoption rates, indicating the power of targeted promotions. Demographic factors such as age and gender also influence adoption. Younger caregivers are more likely to adopt these services, and female caregivers show higher adoption rates compared to their male counterparts. Urban caregivers tend to adopt these services more than those in rural areas, likely due to better internet infrastructure and services in urban areas. More hours spent online per day correlate with higher adoption, as these caregivers are more accustomed to using internet services.

## 4 Discussion

The findings from this study provide important insights into the sociodemographic, economic, and cultural factors influencing the adoption of internet‐based services among female family caregivers in Bangladesh, along with clear implications for enhancing this adoption. To improve the situation, interventions are needed at both societal and policy levels.

From a societal perspective, several factors emerged as critical in shaping caregivers' willingness to adopt these services. Support from family members was a key factor, underscoring the importance of creating a supportive environment where caregivers feel encouraged to explore digital tools. This finding aligns with previous studies indicating that social influence plays a significant role in technology adoption, particularly in collectivist societies where family approval is essential for behavioral change [30, 35]. In Bangladesh, caregivers often rely on traditional caregiving practices passed down through generations, which can create resistance to adopting new digital solutions. Raising awareness within family networks about the benefits of internet‐based services, particularly through community advocacy programs, could accelerate adoption. In Bangladesh, where family structures are central to decision‐making, caregivers are more likely to adopt digital tools if they are endorsed by their immediate family and peers. Promoting the benefits of internet‐based services through family networks and social influencers could increase adoption rates. In addition, caregivers who expressed greater confidence in the reliability of these services were more likely to adopt them. However, concerns about misinformation and the credibility of online caregiving platforms remain barriers. Initiatives that provide caregivers with access to government‐endorsed or healthcare‐approved digital tools could help mitigate these concerns. This highlights the need for trustworthy, dependable platforms that caregivers can rely on, particularly for solving emergency issues, which was identified as a major driver of adoption.

Digital literacy and accessibility also play vital roles. Caregivers with higher internet literacy and easier access to the internet are more inclined to use these services. To address this, community‐based digital literacy programs tailored to caregivers could provide the hands‐on experience needed to build confidence in navigating online resources. Similar initiatives in India and Indonesia have shown that localized digital literacy programs can significantly increase adoption rates, particularly among women in rural communities [18]. However, digital literacy alone is not enough—caregivers also need practical training on how to integrate these tools into daily caregiving tasks. Hands‐on workshops and peer‐led support groups could enhance adoption by providing real‐world use cases and troubleshooting assistance. Additionally, e‐health literacy has been linked to better health outcomes, reinforcing the importance of targeted education programs [21]. Special focus should be placed on reaching caregivers in rural areas, where internet access is limited. Expanding infrastructure to provide reliable, affordable internet is essential to reduce the urban–rural divide in adoption rates. Furthermore, digital literacy programs should incorporate training on online safety and data privacy, as many caregivers expressed concerns about scams and potential misuse of their personal or financial information. Addressing these fears through awareness initiatives and user education could improve trust and adoption.

At the policy level, government support in shaping internet usage policies can have a significant positive impact on adoption. Policies that make internet services more affordable, particularly for low‐income households, would increase access for caregivers across different economic backgrounds. Economic factors, such as household income, were found to influence adoption, with higher‐income caregivers more likely to use internet‐based services. Financial subsidies or discounts on internet services for low‐income caregivers could help bridge this gap, ensuring equal access to vital caregiving tools. Countries such as South Korea and Estonia have successfully implemented subsidized internet programs to enhance digital inclusion [47]. Bangladesh could implement a similar model by offering tiered pricing for internet services, where lower‐income households receive subsidized rates or free access to caregiving platforms. Additionally, partnerships between the government and private tech firms could lead to the development of caregiving‐specific digital services that are both affordable and localized to caregivers' needs. Implementing similar models in Bangladesh could encourage broader adoption of internet‐based caregiving services, particularly among economically disadvantaged groups.

Furthermore, service reliability and usability were critical factors influencing adoption. Services that are easy to use and designed with caregivers in mind are more likely to gain traction. Providers should prioritize user‐friendly interfaces and ensure that their platforms are not only accessible but also responsive in addressing caregivers' needs. The cost of these services is another significant consideration; affordable internet plans and free or low‐cost caregiving platforms could make digital tools more attractive to a wider range of caregivers. Trust in online healthcare services was another barrier identified in this study, consistent with global research highlighting privacy and security concerns as key deterrents to technology adoption [40]. Many caregivers expressed hesitancy in sharing medical information online due to fears of data breaches or cyber fraud. Regulatory measures that enforce data protection policies, as well as visible security features on caregiving platforms, could help build trust and encourage adoption [64, 65]. Addressing these concerns through improved cybersecurity measures and transparent data protection policies can enhance trust and encourage greater adoption.

Promotions targeting female caregivers, particularly those in urban areas, have also been shown to be effective in increasing adoption. Caregivers in urban settings tend to have better access to internet infrastructure, making it easier for them to use these services. In contrast, caregivers in rural areas are at a disadvantage due to poorer infrastructure. Therefore, targeted promotional campaigns that emphasize the benefits of these services, particularly their problem‐solving capabilities and ease of use, could further enhance adoption.

Finally, demographic factors such as age, gender, and time spent online also influence adoption. Younger caregivers, and particularly female caregivers, are more likely to adopt internet‐based services, while older caregivers may face more barriers. This points to the need for age‐specific training and support programs to ensure that older caregivers are not left behind. Gender remains a particularly strong determinant, with patriarchal norms limiting women's access to digital technology in Bangladesh. Previous research has shown that women in low‐income countries face restrictions on independent internet use, often relying on male family members to access online services [50, 66]. This aligns with our findings that many female caregivers, particularly in rural areas, lack personal access to digital devices, making it difficult for them to use internet‐based caregiving tools. Addressing this structural barrier requires gender‐sensitive policies that promote digital equity through increased access to mobile devices and internet connectivity tailored for female users. Additionally, policymakers should consider mobile‐based caregiving applications that require minimal data usage, as affordability constraints remain a major barrier for low‐income women. Moreover, caregivers who spend more time online are more comfortable using digital tools, suggesting that increasing general internet engagement among caregivers could indirectly boost adoption of these services.

### 4.1 Policy Recommendations and Implementation Challenges

The findings from this study highlight the need for policy interventions to enhance the adoption of internet‐based services among female caregivers in Dhaka, Bangladesh. While various policy measures have been proposed, their feasibility, sustainability, and effectiveness require careful consideration. This section outlines key policy recommendations and addresses potential implementation challenges to ensure a practical and impactful approach.

### 4.2 Subsidized Internet and Digital Affordability

Affordability remains one of the strongest barriers to digital adoption among caregivers. Implementing subsidized internet plans specifically for caregiving purposes could help bridge this gap. Countries like South Korea and Estonia have successfully implemented government‐funded internet subsidies, ensuring that vulnerable populations have access to online healthcare services.

#### 4.2.1 Implementation Challenges and Solutions

*Funding Constraints:* Sustaining long‐term subsidies requires significant financial resources. Public–private partnerships between the government, telecom providers, and digital health platforms could enable cost‐sharing models, reducing the financial burden on the state while ensuring affordability.

*Limited Awareness:* Many caregivers may not be aware of available subsidies. Targeted digital awareness campaigns, leveraging community health centers and women's organizations, could improve outreach and participation.

### 4.3 Expansion of Digital Literacy and Caregiver Training Programs

While digital literacy programs exist, many caregivers struggle to apply their skills in real caregiving situations. Integrating practical caregiving‐focused digital training into existing community health programs could enhance adoption. Governments should collaborate with local NGOs and healthcare providers to offer hands‐on workshops, focusing on mobile‐based caregiving tools, online safety, and data privacy.

#### 4.3.1 Implementation Challenges and Solutions

*Sustained Engagement:* Many digital training programs suffer from low long‐term participation. Integrating ongoing refresher training and peer‐led support groups could help caregivers retain skills and confidence.

*Accessibility Barriers:* Some caregivers may find it challenging to attend in‐person training. Providing hybrid learning models, including short video tutorials, app‐based guidance, and SMS‐based training, could improve accessibility.

### 4.4 Strengthening Trust and Online Security Measures

Trust in online caregiving services remains a major concern, especially regarding privacy, misinformation, and potential cyber risks. Many caregivers hesitate to share medical or financial information online due to fear of data breaches. Implementing government‐certified caregiving platforms and mandatory data protection policies could help enhance trust in digital caregiving tools.

#### 4.4.1 Implementation Challenges and Solutions

*Misinformation and Online Fraud:* Many caregivers are skeptical of unverified digital platforms. A government‐endorsed verification system for caregiving apps could improve credibility.

*Lack of Clarity on Data Privacy:* Many caregivers are unaware of how their data are stored and used. Enforcing transparent terms of service, simplified privacy policies, and explicit consent mechanisms could increase trust and adoption.

### 4.5 Affordable and User‐Friendly Caregiving Platforms

Beyond internet affordability, the cost and complexity of caregiving applications also pose barriers to adoption. Many digital health platforms require subscription fees or complex interfaces, limiting accessibility for caregivers with limited digital exposure.

#### 4.5.1 Implementation Challenges and Solutions

*High Subscription Costs:* Some caregiving platforms require premium memberships, restricting access. Developing government‐funded or low‐cost caregiving apps with freemium models could improve accessibility.

*Complex User Interfaces:* Many caregivers, particularly those new to digital tools, struggle with complicated app navigation. Simplifying app design with localized language support, voice‐based navigation, and AI‐driven assistance could enhance usability.

### 4.6 Gender‐Sensitive Digital Policies

Given the gendered nature of caregiving, policies should prioritize female caregivers' digital access and address structural barriers that limit their engagement with technology. Policies should include targeted financial assistance for women, mobile‐first caregiving solutions, and digital inclusion programs tailored for female caregivers.

#### 4.6.1 Implementation Challenges and Solutions

*Household‐Level Restrictions on Digital Access:* Many female caregivers share devices with family members, limiting their ability to use caregiving apps independently. Incentivizing mobile ownership for caregivers through financial aid or micro‐loan programs could improve access.

*Limited Representation of Women in Digital Health Initiatives:* Many digital health programs do not cater specifically to female caregivers' needs. Ensuring women‐centered technology design and policymaking could lead to higher adoption rates.

## 5 Limitations

While this study provides valuable insights, several limitations should be acknowledged. The research was conducted exclusively in Dhaka, Bangladesh, limiting the generalizability of findings to rural and semi‐urban areas where internet access, infrastructure, and sociocultural dynamics differ. Expanding the survey to a broader geographic scope was constrained by resource limitations, logistical challenges, and difficulties in reaching caregivers in remote areas. Future studies should include participants from diverse regions to provide a more comprehensive perspective. Cross‐sectional design captures internet adoption behaviors at a single point in time, preventing an assessment of long‐term trends and policy‐driven changes. A longitudinal approach would offer deeper insights into how caregivers' digital engagement evolves over time. This study relied on self‐reported data, which may introduce recall and social desirability bias in responses about internet usage and digital literacy. Future research could integrate objective data sources such as platform usage logs to improve reliability. Additionally, many female caregivers in Bangladesh share digital devices with family members, limiting their privacy and independent access to online caregiving tools. This study did not directly measure device ownership or intra‐household digital control, which should be explored in future research. Despite these limitations, this study offers crucial contributions to understanding the sociodemographic, economic, and cultural barriers affecting internet‐based service adoption among female caregivers. Addressing these gaps in future research will further support efforts to enhance digital inclusivity and caregiving technology solutions in Bangladesh and similar contexts.

## 6 Conclusion

Improving the adoption of internet‐based services among female family caregivers in Bangladesh will require a multifaceted approach. At the societal level, enhancing family support, building digital literacy, and promoting the benefits of these services through trusted networks can encourage broader use. At the policy level, expanding internet access, improving service affordability, and ensuring reliability and usability are essential steps toward making digital services more accessible to all caregivers. By addressing both societal and policy‐level barriers, Bangladesh can better support its caregivers, improving their ability to provide high‐quality care with internet‐based tools. Future research should build on these insights by exploring diverse populations and longitudinal effects, as well as evaluating specific interventions aimed at boosting digital service adoption. Addressing these areas will help create a more supportive environment for female caregivers, ensuring they have the tools and resources needed to manage their caregiving responsibilities effectively.

## Author Contributions

**Mohammad Ishtiaque Rahman:** conceptualization, writing – original draft, methodology, visualization, formal analysis. **Jahangir Alam:** investigation, methodology, supervision, project administration. **Khadija Khanom:** writing – review and editing, methodology. **Forhan Bin Emdad:** writing – original draft, validation, writing – review and editing.

## Ethics Statement

The study was approved by the Center for Project Management and Information Systems, University of Dhaka Institutional Review Board (Protocol ID: 20240314).

## Conflicts of Interest

The authors declare no conflicts of interest.

## Transparency Statement

The lead author Jahangir Alam affirms that this manuscript is an honest, accurate, and transparent account of the study being reported; that no important aspects of the study have been omitted; and that any discrepancies from the study as planned (and, if relevant, registered) have been explained.

## Data Availability

Anonymized versions of the data that support the findings of this study are available from the corresponding author upon reasonable request. Access is subject to approval by the Institutional Review Board and compliance with privacy regulations.
